# Genetic stability of *Brucella abortus* isolates from an outbreak by multiple-locus variable-number tandem repeat analysis (MLVA16)

**DOI:** 10.1186/1471-2180-14-186

**Published:** 2014-07-11

**Authors:** Elaine Maria Seles Dorneles, Jordana Almeida Santana, Telma Maria Alves, Rebeca Barbosa Pauletti, Juliana Pinto da Silva Mol, Marcos Bryan Heinemann, Andrey Pereira Lage

**Affiliations:** 1 Laboratório de Bacteriologia Aplicada, Departamento de Medicina Veterinária Preventiva, Escola de Veterinária, Universidade Federal de Minas Gerais, Av. Antônio Carlos, 6627, Caixa Postal 567, 31270-901 Belo Horizonte, MG, Brazil; 2 Present address: Departamento de Medicina Veterinária Preventiva e Saúde Animal - VPS, Faculdade de Medicina Veterinária e Zootecnia da Universidade de São Paulo, Av. Prof. Dr. Orlando Marques de Paiva, 87 - Cidade Universitária, 05508-270 São Paulo, SP, Brazil

**Keywords:** Genotyping, MLVA16 stability, Bovine brucellosis, *B. abortus*

## Abstract

**Background:**

Brucellosis caused by *Brucella abortus* is one of the most important zoonoses in the world. Multiple-locus variable-number tandem repeat analysis (MLVA16) has been shown be a useful tool to epidemiological traceback studies in *B. abortus* infection. Thus, the present study aimed (*i*) to evaluate the genetic diversity of *B. abortus* isolates from a brucellosis outbreak, and (*ii*) to investigate the in vivo stability of the MLVA16 markers.

**Results:**

Three-hundred and seventy-five clinical samples, including 275 vaginal swabs and 100 milk samples, were cultured from a brucellosis outbreak in a cattle herd, which adopted RB51 vaccination and test-and-slaughter policies. Thirty-seven *B. abortus* isolates were obtained, eight from milk and twenty-nine from post-partum/abortion vaginal swabs, which were submitted to biotyping and genotyping by MLVA16. Twelve *B. abortus* isolates obtained from vaginal swabs were identified as RB51. Twenty four isolates, seven obtained from milk samples and seventeen from vaginal swabs, were identified as *B. abortus* biovar 3, while one isolate from vaginal swabs was identified as *B. abortus* biovar 1. Three distinct genotypes were observed during the brucellosis outbreak: RB observed in all isolates identified as RB51; W observed in all *B. abortus* biovar 3 isolates; and Z observed in the single *B. abortus* biovar 1 isolate. Epidemiological and molecular data show that the *B. abortus* biovar 1 genotype Z strain is not related to the *B. abortus* biovar 3 genotype W isolates, and represents a new introduction *B. abortus* during the outbreak.

**Conclusions:**

The results of the present study on typing of multiple clinical *B. abortus* isolates from the same outbreak over a sixteen month period indicate the *in vivo* stability of MLVA16 markers, a low genetic diversity among *B. abortus* isolates and the usefulness of MLVA16 for epidemiological studies of bovine brucellosis.

## Background

Brucellosis is a bacterial disease caused by microorganisms of the genus *Brucella* that affects different livestock and wild animal species, besides man [1]. Cattle are the preferred host of *Brucella abortus* and the economic importance attributed to bovine brucellosis is based on losses caused by abortions, stillbirths, weight loss, decreased milk production and the establishment of sanitary barriers to international trade of animals and their products [1].

Because direct and indirect losses triggered by *B. abortus* infection, the control and eradication of bovine brucellosis is an important goal of several countries where the disease is endemic, including Brazil that since 2001 has a national program on the control and eradication of brucellosis and tuberculosis (Programa Nacional de Controle e Erradicação da Brucelose e Tuberculose – PNCEBT) [2,3].

Typing of *Brucella* spp. by biovar determination and genotyping are important tools in a brucellosis control and eradication programs [4]. Indeed, molecular typing methods are commonly used to investigate epidemiological relationships among isolates and sources of infection [5]. In this sense, the multiple locus variable number of tandem repeats (VNTR) analysis (MLVA) has proved to be an important tool in molecular epidemiology studies of brucellosis [6-11], in the characterization of new *Brucella* species [12] and in the evaluation of sources of human infection [13].

In MLVA context, the assessment of the genetic stability is one of the vital elements to guarantee the successful use of this typing method, since it could confirm its ability to differentiate between two independent strains, while depicting relationships among strains from the same source [14]. The genetic stability can be deduced by typing results of multiple isolates originating from the same strain or strains after numerous passages [8,9,15,16]. In *in vitro* evaluations, *B. abortus* vaccine and field strains have been shown to be stable by MLVA16 [9,15-17]. Regarding to *in vivo* stability of the MLVA16 markers, data based on *B. abortus* experimental infection showed only few changes in the hyper-variable loci [9]. Whereas, MLVA16 *in vivo* stability have not been evaluated under natural conditions.

Therefore, the aims of this study were (*i*) to evaluate the genetic diversity of *B. abortus* isolates obtained from a single cattle brucellosis outbreak and (*ii*) to investigate the *in vivo* stability of the MLVA16 markers.

## Results

### Serologic testing

**Figure 1:**
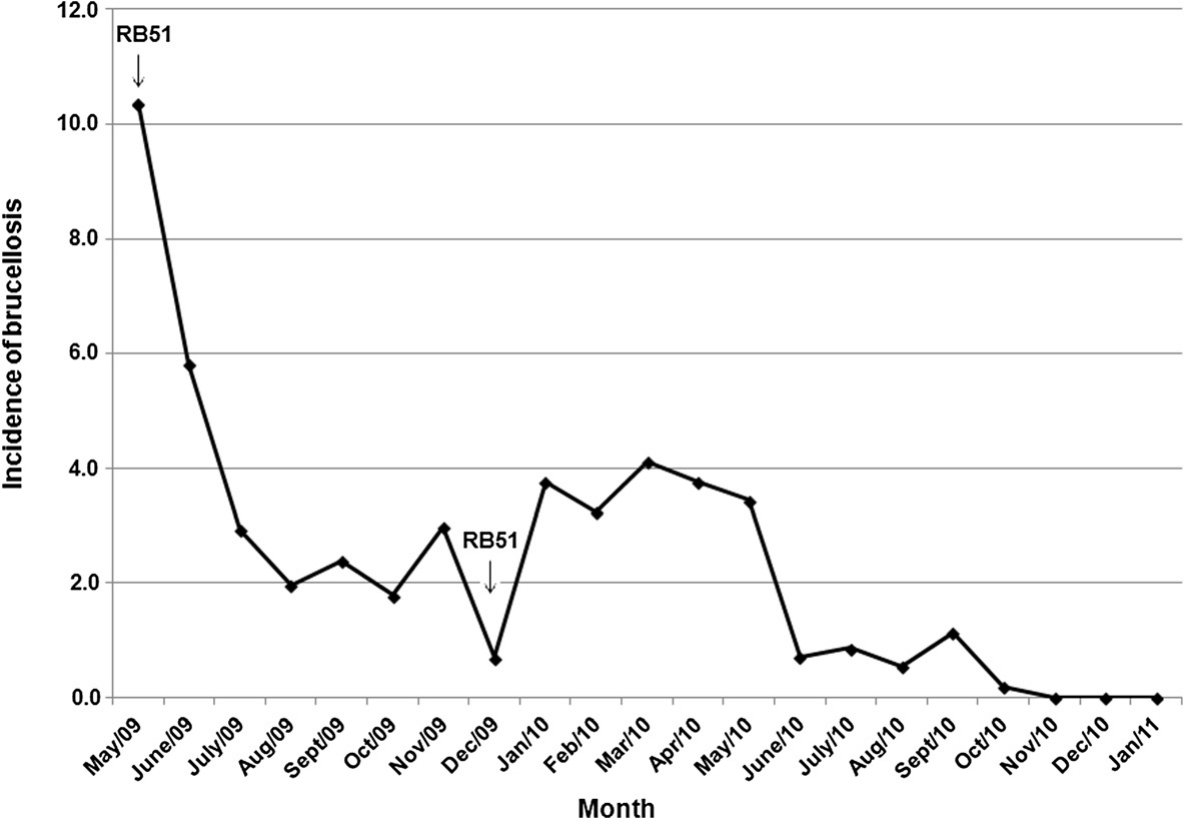
**Monthly incidence of serologically positive cattle in a bovine brucellosis outbreak from May 2009 to January 2011, Minas Gerais, Brazil.** All cattle older than 24 months were monthly tested for brucellosis [Rose Bengal Plate Agglutination Test (RBPAT) as a screening test and the Standard Tube Agglutination Test (STAT) and 2-Mercaptoethanol Test (2ME) as a confirmatory test]. Positive animals were culled until three consecutive negative herd results were obtained. The herd was vaccinated with RB51 on May 2009 and on December 2009 (RB51 vaccinations are highlighted with arrows).

The incidence of brucellosis in the herd between May 2009 and January 2011 is shown in Figure 1. No positive animal was observed on three monthly tests after October 2011.

### Isolation and identification

Thirty-seven isolates of *B. abortus* were obtained from 340 cows (375 samples) during the 16 months period of outbreak: eight from milk samples (8% - 8/100) and twenty-nine from post-partum vaginal swabs (10.5% - 29/275) (Table 1). Of the *B. abortus* isolates obtained from vaginal swabs samples eight were associated to abortion/stillborn occurrence*.* Among animals that had vaginal swabs and milk samples collected (35 animals) three *B. abortus* isolates were obtained, all from vaginal swabs.

**Table 1:** **Clinical signs, biotyping, molecular identification and genotyping of *B. abortus *** **isolates from a bovine brucellosis outbreak**

**Strain**	**Clinical sample**	**Collection date**	**Biovar**	**Genotype**^**b**^	**Clinical signal**
**1414**	vaginal swab	09/01/2009	3	W	Abortion
**2757**	vaginal swab	09/01/2009	1	RB	-
**2985**	vaginal swab	09/01/2009	3	W	-
**3144**	vaginal swab	10/01/2009	1	RB	-
**2575**	vaginal swab	09/02/2009	3	W	-
**2558**	vaginal swab	07/03/2009	1	RB	-
**2608**	vaginal swab	08/03/2009	1	RB	-
**1379**	vaginal swab	07/04/2009	1	RB	-
**3136**	vaginal swab	10/05/2009	1	RB	-
**2634**	vaginal swab	10/07/2009	3	W	Abortion
**1365**	milk	07/27/2009	3	W	Stillbirth
**2476**	milk	07/27/2009	3	W	-
**2598**	milk	07/27/2009	3	W	Stillbirth
**2683**	milk	07/27/2009	3	W	-
**2785**	milk	07/27/2009	3	W	-
**2965**	milk	07/27/2009	3	W	-
**2980**	milk	07/27/2009	3	W	-
**3159**	vaginal swab	07/27/2009	1	RB	-
**2922**	vaginal swab	08/18/2009	1	RB	-
**3169**	vaginal swab	08/21/2009	1	RB	-
**2545**	vaginal swab	09/19/2009	3	W	Stillbirth
**2595**	vaginal swab	10/17/2009	3	W	-
**1198**	vaginal swab	10/28/2009	3	W	Abortion
**1219**	vaginal swab	10/28/2009	3	W	Abortion
**2251**	vaginal swab	07/11/2009	3	W	-
**2496**	vaginal swab	07/11/2009	3	W	-
**2700**	vaginal swab	09/11/2009	3	W	Abortion
**2588**	vaginal swab	01/28/2010	3	W	-
**3238**	vaginal swab	05/03/2010	3	W	Stillbirth
**2726**	vaginal swab	03/28/2010	1	RB	-
**3006**	vaginal swab	05/04/2010	3	W	-
**2998**	vaginal swab	04/15/2010	3	W	-
**3022**	vaginal swab	04/27/2010	3	W	-
**3016**	vaginal swab	04/30/2010	3	W	-
**2774**	vaginal swab	02/06/2010	1^a^	Z	-
**2543**	vaginal swab	03/11/2010	1	RB	-
**2850**	vaginal swab	03/11/2010	1	RB	-

^a^*B. abortus* biovar 1field strain.

^b^MLVA16 genotype.

Based on phenotypic characteristics, twenty four strains were classified as *B. abortus* biovar 3 (seven from milk and seventeen from vaginal swabs) and thirteen as *B. abortus* biovar 1 (one from milk and twelve from vaginal swabs) (Table 1). All strains classified as *B. abortus* biovar 3 presented smooth colonial morphology. Among the thirteen *B. abortus* biovar 1 strains, twelve exhibited rough colonial morphology and growth on tryptose agar plates supplemented with rifampicin (200 μg/mL) (all from vaginal swabs), whereas one strain isolated from a milk sample was classified as smooth *Brucella* spp.

All strains were confirmed as belonging to the genus *Brucella* by genus-specific PCR [18], and as *B. abortus* by AMOS-enhanced PCR and Bruce-ladder multiplex PCR [19,20]. Moreover, the twelve isolates phenotypically classified as *B. abortus* biovar 1 rough strains were confirmed as RB51 vaccine strain by AMOS-enhanced PCR [19].

### MLVA16

The set of genetic markers which comprises the MLVA16 was observed to be stable *in vivo*, even when evaluated from different clinical specimens over a 16 months outbreak. Analysis of the MLVA16 loci revealed three distinct genotypes among the 37 *B. abortus* strains isolated during the brucellosis outbreak, herein these genotypic patterns were labeled RB, W and Z, for new genotypes the identification adopted was sequential in the same way used by Minharro et al. [11] (Table 1). The genotype RB was observed in all twelve strains identified as *B. abortus* RB51, which were all obtained from vaginal swab samples. The genotype W was observed in all twenty four isolates classified as *B. abortus* biovar 3 (seven from milk and seventeen from vaginal swabs), while genotypic profile Z was observed in only one strain, which was isolated from milk and identified as a smooth *B. abortus* biovar 1 field strain. This biovar 1 strain was isolated eleven months after confirmation of brucellosis in the herd on February/2010, being one of the last strains isolated, since clinical samples were collected until May/2010 (Table 1).

Patterns obtained in the sixteen VNTR loci are summarized in Table 2. Comparison of the results observed in the eight conserved loci included in panel 1 by Le Flèche et al. [6] with those available in the MLVA bank 2014 (http://mlva.u-psud.fr/brucella/) showed genotypes 27, 40 and 28 for the strains classified as MLVA16 genotypes RB, W and Z, respectively. Genotype RB was identical to the previously describe [6,16] for RB51 vaccine strain. The MLVA16 pattern for genotypes W and Z did not find correspondence with those deposited on MLVA bank 2014. In comparison with genotype W, the genotype Z exhibited different alleles in the loci Bruce04, Bruce06, Bruce11, Bruce16, Bruce18 and Bruce30 (Table 2).

**Table 2:** **Allelic types of MLVA16 loci observed for *B. abortus *** **strains isolated from a bovine brucellosis outbreak**

**Locus**	**Tandem repeat copy numbers**			**Number of alleles**	**Panel**
	**Genotype W**	**Genotype Z**	**Genotype RB**		
Bruce06	3	4	4	2	1
Bruce08	5	5	5	1	1
Bruce11	3	4	4	2	1
Bruce12	12	12	12	1	1
Bruce42	2	2	2	1	1
Bruce43	2	2	3	2	1
Bruce45	3	3	3	1	1
Bruce55	3	3	3	1	1
Bruce18	7	6	6	2	2A
Bruce19	42	42	42	1	2A
Bruce21	8	8	8	1	2A
Bruce04	5	3	3	2	2B
Bruce07	4	4	7	2	2B
Bruce09	3	3	3	1	2B
Bruce16	3	5	3	2	2B
Bruce30	3	6	5	3	2B

## Discussion

Results from the present study on 37 *B. abortus* isolates from milk and vaginal swabs taken from 340 cows (375 samples) during a 16 months period showed the *in vivo* genetic stability of the MLVA16 markers. Molecular typing methods are commonly used to investigate epidemiological relationships among isolates and sources of infection [5]. However, before being used for those purposes, PCR methods for molecular typing require careful in-house validation of typeability, reproducibility, repeatability, stability, discriminatory power and epidemiologic concordance [5,21]. The findings of this study, associated with previous data on the high discriminatory power and epidemiologic concordance of MLVA16, besides its good typeability and *in vitro* stability [6,7,9-11,13,15-17], corroborate the use of MLVA16 as suitable typing method for refining the understanding of the epidemiology of bovine brucellosis.

Our results showed a low genetic diversity and the existence of three different *B. abortus* strains within a focus of bovine brucellosis, the RB51 vaccine strain (genotype RB), recovered from animals that were twice vaccinated in the period, and two field strains (genotypes W and Z).

In the present study no RB51 was isolated from the milk of any animal during the post-partum period (30–60) but only from vaginal swabs, although some cows have been vaccinated in the last third of pregnancy and a highly sensitive diagnostic strategy have been employed [22]. These results were corroborated by previous studies that also reported no recovery of *B. abortus* RB51 from any of the milk sample tested by conventional bacteriological methods of cows vaccinated during pregnancy or 30–60 days after delivery [23,24]. Regarding the presence of viable RB51 in postpartum vaginal secretion, it is important to consider that all animals from which RB51 were recovered after delivery were over 5 months of gestation by the time of revaccination with this strain and that no abortion was related to RB51 isolation (Table 1). Thus, it is likely that the RB51 booster had contributed to the recovery of the vaccine strain in postpartum period, since it has been observed that during transition period a depression in cell-mediated response occurs, which leads to a decrease of the resistance to disease or increase of the residual virulence of vaccines [25].

Other important finding of this study was the demonstration of viable *B. abortus* in milk from infected animals associated with the outbreak, corroborating the public health risk of the consumption of raw milk and unpasteurized dairy products [26]. The colonization of the mammary gland and associated lymph nodes by *B. abortus* with excretion of microorganisms in milk was already demonstrated [27]. In addition, it is important to emphasize that the methodologies used for bacterial culture and molecular identification were able to differentiate RB51 vaccine strain from *B. abortus* field strains.

Interestingly, field strains isolated from this brucellosis outbreak showed distinct genotypes and biotypes, suggesting that the outbreak had two different sources of infection. The majority of *B. abortus* isolates from the outbreak (24/25) were classified as *B. abortus* biovar 3 genotype W, which remained the single cause of brucellosis in the herd for eleven months (from March 2009 to February 2010). This widely demonstrates that the introduction of this strain in the herd was responsible for the occurrence of the outbreak and consequently for the high abortion rate observed at the end of 2008. Furthermore, in February 2010, the introduction into the herd of another field strain, *B. abortus* biovar 1 genotype Z, was observed. This genotype Z was confirmed by a twice repetition of MLVA16 genotyping assay that showed the same results. There are two possible explanations for the presence of this infected animal, negative to the conventional serological tests adopted in the herd: first, this heifer was congenitally and persistently infected without seroconversion [28], and second, the negative serological result from this infected heifer was a false negative result inherent to any diagnostic test. However, the diagnostic strategy employed was very sensitive [29], which tends to minimized false negative results. Since this second field strain was represented by just one isolate, it is very likely that the control policy adopted in the herd, which included test-and-removal procedures, has prevented the dissemination of this strain to other animals and consequently a new increase in the brucellosis prevalence levels into the herd.

Besides the differences in the classification of biovar, the differences observed between the genotypes of the field strains were very large and not limited to differences in the more variable loci (Bruce04, Bruce16, Bruce18 and Bruce30), but were also observed in conserved loci, such as Bruce06 and Bruce11 (Table 2). Therefore, the identification of the new source of infection in the herd was only possible due the use of MLVA16, otherwise the genotype Z strain would have been considered just another *B. abortus* isolate within the outbreak.

Epidemiological data of the herd also confirmed that the *B abortus* biovar 1 genotype Z strain was a newly introduced strain in the farm, since the young heifer from which this strain was isolated was introduced in the herd shortly at the end of the outbreak (November/2009). In fact, it has been widely demonstrated that the purchase of infected animals is the main risk factor for the introduction of brucellosis in free herds [30]. Unfortunately, it was not possible to obtain more epidemiological data about the heifer from which the second *B. abortus* field strain was isolated, such as the brucellosis status of the herd of origin, which would have allowed a better understanding about the epidemiology of the genotype Z strain.

The MLVA16 panel 1 profiles of both *B. abortus* isolates, genotypes 28 and 40 by the MLVAbank, have already been previously observed in Minas Gerais State [11]. Moreover, the comparison between MLVA16 genotypes of *B. abortus* biovar 3 field strain (W) and those previously described by Minharro et al. [11] revealed differences restricted to hypervariable loci [panels 2A (Bruce19) and 2B (Bruce04)], suggesting a possible epidemiological link between these strains, since all were isolated in the state of Minas Gerais and were also identified as biovar 3. For the single field strain classified as genotype Z, the comparison with MLVA16 patterns previously described by Minharro et al. [11] showed a genetic distance of one locus (Bruce19) from a *B. abortus* biovar 2 also isolated from Minas Gerais State.

In a brucellosis control and eradication program the use of an accurate surveillance and highly discriminatory typing method is essential to characterize an outbreak and determine the source of infection and the transmission routes. The present results on typing multiple *B. abortus* isolates from an outbreak originated from an outbreak in the same herd depicted the *in vivo* stability of the MLVA16 markers. The set of loci that comprise the MLVA16 demonstrated to be very stable, even when assessed over the time span of one year and four months, since the field strain mainly responsible by the outbreak (W) and the RB51 vaccine strain recovered from vaccinated animals showed unchanged MLVA16 profiles. These findings are extremely important because they definitely confirm the ability of the MLVA16 to establish correct epidemiological correlations, since, besides having a high discriminatory power, MLVA16 markers were also stable under natural selection pressure exerted by the host, during the sixteen months assessed. Thus, these results increase the confidence in the traceback established from the results of MLVA16 and further support this technique as the choice one for typing *B. abortus*.

Futhermore, the examination of *in vitro* stability of the *B. abortus* RB51 vaccine strain, *B. abortus* strain 2308 and *B. abortus* field isolates by serial passages in culture medium showed no change in MLVA16 profile [9,16]. Likewise, the analysis of *B. abortus* S19 vaccine strain from different batches of different manufacturers did not reveal significant differences in MLVA16 pattern [17]. Concerning the evaluation of *in vivo* genetic stability of MLVA16 loci*,* it has also been demonstrated that passage of *B. abortus* RB51 in cattle and of *B. abortus* 2308 in mouse did not lead to changes in any marker of MLVA16 [9]. Nevertheless, minor changes in VNTR pattern were observed in *in vitro* passages of *B. abortus* 544 and in genotyping of multiple *B. abortus* isolates from the same outbreak [9]. Her et al. [9] found different allelic profiles in seven of twenty-three herds in which more than one *B. abortus* isolates were obtained. Those different genotypes from same outbreak showed mutations only in the loci Bruce 30 and 43, which did not seem to affect the identification of a possible common origin of the strains [9]. However, our data showing the high *in vivo* stability of the MLVA16 loci have as main findings over previous data the large numbers of isolates (37) obtained from the same source, the long period of time assessed (16 months) and the typing of strains colonizing different sites (mammary gland or reproductive tract), and therefore under different selective environmental pressures.

## Conclusions

The results of the present study on typing of multiple isolates from different clinical specimens originating from the same outbreak over a sixteen month period indicate the *in vivo* stability of the MLVA16 markers, a low genetic diversity among *B. abortus* isolates and the usefulness of MLVA16 for epidemiological studies of bovine brucellosis.

## Methods

### Outbreak description

The experiment was conducted during an outbreak of brucellosis in a cattle herd located in Matozinhos, Minas Gerais, Brazil. The herd was composed of 705 Holstein dairy cows, reared in an intensive system. All animals in the herd were vaccinated with S19 between 3 to 8 month of age and tested for brucellosis twice a year according to PNCEBT [3] [Rose Bengal Plate Agglutination Test (RBPAT) as a screening test and the Standard Tube Agglutination Test (STAT) and 2-Mercaptoethanol Test (2ME) as a confirmatory tests]. During 2008, several lots of heifers were acquired in order to increase the milk production of the herd. Prior to introduction into the herd, all heifers were serologically tested [3] and only lots with brucellosis-negative animals were acquired.

Suspicion of brucellosis in the herd started with an abnormal high abortion rate (15%) observed by the end of 2008. Brucellosis was confirmed serologically on March 2009, and a control program based on mass vaccination with RB51 and a test-and-slaughter policy was initiated. PNCEBT demands the compulsory S19 vaccination of female calves aged 3 to 8 month and recommends the voluntary RB51 vaccination of heifers older than 8 month and cows in *B. abortus* infected herds. All female animals over eight months of age were revaccinated with RB51, including pregnant heifers and cows. The herd was revaccinated with RB51 on two different occasions: the first in May 2009 and the second in December 2009. Moreover, all cattle older than 24 months were monthly tested for brucellosis (RBPAT as screening test and STAT and 2ME as a confirmatory test) and the positive animals were culled [3] until three consecutive negative herd results were obtained.

The experimental protocol was approved by Ethics Committee on Animal Experimentation of Universidade Federal de Minas Gerais (CETEA/UFMG - Protocol 139/10).

### Clinical samples

Three-hundred and seventy-five clinical samples were collected from 340 cows between January 2009 and May 2010 during the brucellosis outbreak; those included 275 vaginal swabs and 100 milk samples. Of thirty-five animals were collected both, vaginal swabs and milk samples. Vaginal swabs were collected in Stuart medium from all cows immediately after abortion (twenty-two samples) or delivery, whereas milk samples were obtained from all cows that had 60 days or less of the last parturition or abortion at 57 days after the first RB51 vaccination (07/27/2009). For each animal, 50 mL of milk was collected after discarding of the first jet (milk from the four quarter was mixed). Both clinical samples were stored at -20°C until processing.

### Culture conditions

Vaginal swabs were thawed and directly plated onto duplicate tryptose agar plates (Difco, USA) with antibiotics (Farrell’s selective supplement) (Oxoid, UK) [31], and they were also inoculated in 10 mL of enrichment medium (tryptose broth supplemented with Farrell’s selective supplement) [22]. Milk samples were thawed and centrifuged at 2500 × *g*, for 15 minutes. The intermediated phase was discarded and the supernatant was mixed with the pellet. Aliquots of 100 μL of each mixture was immediately inoculated onto duplicate tryptose agar plates (Difco, USA) with antibiotics (Farrell’s selective supplement) (Oxoid, UK) [31]. Another aliquot of 1 mL of each mixture was diluted in 9 mL of enrichment medium. The enrichment media inoculated with vaginal swabs or milk were incubated at 37°C for seven days in 5% of CO_2_ and then inoculated onto tryptose agar plates with antibiotics [22]. All plates were incubated in 5% of CO_2_ at 37°C for at least 14 days [31].

### Identification and biotyping of *Brucella* spp. isolates

*Brucella* spp. solates were identified to genus based on colony morphology, positive tests for urease, catalase and nitrate reduction, and negative tests for motility and citrate utilization [31,32]. Classification into species and biovar was performed by H_2_S production, agglutination with sera anti-A, anti-M, anti-R and acriflavin, growth in CO_2_ and O_2_ atmospheres and sensitivity to thionin (20 μg/mL and 40 μg/mL), basic fuchsin (20 μg/mL), and rifampicin (200 μg/mL) [31-33].

Suspected *Brucella* spp. colonies were also suspended in 100 μL of Tris EDTA (10 mM Tris–HCl, 1 mM EDTA, pH 8.0), inactivated at 85°C for 2 hours and subjected to genomic DNA extraction [34]. DNA quality and concentration were determined by spectrophotometry [35].

Polymerase chain reaction (PCR) for amplification of the gene *bcsp31*[18] and AMOS-enhanced PCR [19] were also used for identification of isolates [see Additional file 1: Table S1]. Since *B. abortus* biovar 3 strains are not identified by AMOS-enhanced PCR, these strains were also analyzed by Bruce-ladder multiplex PCR [20] to confirm their identification as *B. abortus* [see Additional file 1: Table S1]*.*

Reference strains, used as control in different procedures, were: *B. abortus* biovar 1 544 = ATCC 23448^T^; *B. abortus* biovar 2 ATCC 23449; *B. abortus* biovar 3 Tulya = ATCC 23450; *B. abortus* biovar 4 292 = ATCC 23451; *B. abortus* biovar 5 3196 = ATCC 23452; *B. abortus* biovar 6 870 = ATCC 23453; *B. abortus* biovar 9 C68 = ATCC 23455; *B. abortus* biovar 1 S19; *B. abortus* biovar 1 RB51; *B. melitensis* biovar 1 16 M = ATCC 23456^T^; *B. ovis* Reo 198; *B. suis* biovar 1 1330 = ATCC 23444; *Escherichia coli* = ATCC 25922; *E. coli* B41; *Listeria monocytogenes* = ATCC 19115; *Pseudomonas aeroginosa* = ATCC 27853; *Salmonella enterica* serovar Typhimurium = ATCC 14028; and *Staphylococcus aureus* = ATCC 29213.

### MLVA 16 genotyping

DNA from each strain was genotyped by a subset of 16 tandem repeat loci (MLVA16) as previously described [6,7] [see Additional file 1: Table S1]. The method analyses 16 VNTR loci divided into three panels: panel 1 composed of eight minisatellites (Bruce06, Bruce08, Bruce11, Bruce12, Bruce42, Bruce43, Bruce45 and Bruce55); panel 2A composed of three microsatellites (Bruce18, Bruce19 and Bruce21); and panel 2B with five microsatellites (Bruce04, Bruce07, Bruce09, Bruce16 and Bruce30) [6,7].

The amplified products were submitted to electrophoresis in 2% or 3% agarose gel, for the mini and microsatellites, respectively, in Tris-borate-EDTA 1X (TBE) buffer, stained with 0.5 mg/mL ethidium bromide, visualized under UV light, and photographed (ImageMaster VDS, Phamarcia Biotech, Sweden). DNA size markers 100 bp (100 bp DNA Ladder, New England Biolabs, USA) and 25 bp (25 bp DNA Step Ladder, Promega, USA) were used to estimate the tandem repeat unit length.

Each estimated band size was converted into number of repeat units for each locus, with aid of the software BioNumerics 5.1 (Applied Maths, Belgium), as described [6]. Clustering analysis was performed using the same software based on the category coefficient and the unweighted pair group method with arithmetic mean (UPGMA) algorithm [6].

## Competing interests

The authors declare that they have no competing interests.

## Authors’ contributions

EMSD participated in the design, data acquisition and analysis, and wrote the paper. JAS, TMA, RBP and JPSM participated in data acquisition and analysis. MBH and APL conceived the study, participated in design of the study, data interpretation and reviewed the manuscript. All authors read and approved the final manuscript.

## Supplementary Material

Additional file 1: Table S1 Primers used in the present study. Click here for file
