# Supplementary material for: Genetic stability of Brucella abortus isolates from an outbreak by multiple-locus variable-number tandem repeat analysis (MLVA16)
Source: BMC Microbiol. 2014 Jul 11;14:186. doi: 10.1186/1471-2180-14-186 (PMC4112982; doi:10.1186/1471-2180-14-186)
Supplement: Additional file 1: Table S1 — Primers used in the present study. [file 1471-2180-14-186-S1.pdf]

Additional file 1 - Primers used in the present study.

Primer	Sequence 5' – 3'	Assay	Reference
B4	TGGCTCGGTTGCCAATATCAA	gene *bcsp31*	[18]
B5	CAGACCTTGAAAGGCAAGCGCG	gene *bcsp31*	[18]
B. abortus specific	GACGAACGGAATTTTTCCAATCCC	AMOS-enhanced	[19]
B. melitensis specific	AAATCGCGTCCTTGCTGGTCTGA	AMOS-enhanced	[19]
B. ovis specific	CGGGTTCTGGCACCATCGTCG	AMOS-enhanced	[19]
B. suis specific	GCGCGGTTTTCTGAAGGTTCAGG	AMOS-enhanced	[19]
IS*711* specific	TGCCGATCACTTAAGGGCCTTCAT	AMOS-enhanced	[19]
Eri 1	GCGCCGCGAAGAACTTATCAA	AMOS-enhanced	[19]
Eri 2	CGCCATGTTAGCGGCGGTGA	AMOS-enhanced	[19]
RB51/2308	CCCCGGAAGATATGCTTCGATCC	AMOS-enhanced	[19]
BMEI0998F	ATCCTATTGCCCCGATAAGG	Bruce-ladder	[20]
BMEI0997R	GCTTCGCATTTTCACTGTAGC	Bruce-ladder	[20]
BMEI0535F	GCGCATTCTTCGGTTATGAA	Bruce-ladder	[20]
BMEI0536R	CGCAGGCGAAAACAGCTATAA	Bruce-ladder	[20]
BMEII0843F	TTTACACAGGCAATCCAGCA	Bruce-ladder	[20]
BMEII0844R	GCGTCCAGTTGTTGTTGATG	Bruce-ladder	[20]
BMEI1436F	ACGCAGACGACCTTCGGTAT	Bruce-ladder	[20]
BMEI1435R	TTTATCCATCGCCCTGTCAC	Bruce-ladder	[20]
BMEII0428F	GCCGCTATTATGTGGACTGG	Bruce-ladder	[20]
BMEII0428R	AATGACTTCACGGTCGTTCG	Bruce-ladder	[20]
BR0953F	GGAACACTACGCCACCTTGT	Bruce-ladder	[20]
BR0953R	GATGGAGCAAACGCTGAAG	Bruce-ladder	[20]
BMEI0752F	CAGGCAAACCCTCAGAAGC	Bruce-ladder	[20]
BMEI0752R	GATGTGGTAACGCACACCAA	Bruce-ladder	[20]
BMEII0987F	CGCAGACAGTGACCATCAAA	Bruce-ladder	[20]
BMEII0987R	GTATTCAGCCCCCGTTACCT	Bruce-ladder	[20]
Bruce06-F	ATGGGATGTGGTAGGGTAATCG	MLVA16	[6]
Bruce06-R	GCGTGACAATCGACTTTTTGTC	MLVA16	[6]
Bruce08-F	ATTATTCGCAGGCTCGTGATTC	MLVA16	[6]
Bruce08-R	ACAGAAGGTTTTCCAGCTCGTC	MLVA16	[6]
Bruce11-F	CTGTTGATCTGACCTTGCAACC	MLVA16	[6]
Bruce11-R	CCAGACAACAACCTACGTCCTG	MLVA16	[6]
Bruce12-F	CGGTAAATCAATTGTCCCATGA	MLVA16	[6]
Bruce12-R	GCCCAAGTTCAACAGGAGTTTC	MLVA16	[6]
Bruce42-F	CATCGCCTCAACTATACCGTCA	MLVA16	[6]

Bruce42-R	ACCGCAAAATTTACGCATCG	MLVA16	[6]
Bruce43-F	CATCGCCTCAACTATACCGTCA	MLVA16	[6]
Bruce43-R	ACCGCAAAATTTACGCATCG	MLVA16	[6]
Bruce45-F	ATCCTTGCCTCTCCCTACCAG	MLVA16	[6]
Bruce45-R	CGGGTAAATATCAATGGCTTGG	MLVA16	[6]
Bruce55-F	TCAGGCTGTTTCGTCAGTCTT	MLVA16	[6]
Bruce55-R	AATCTGGCGTTCGAGTTGTTCT	MLVA16	[6]
Bruce04-F	TGACGAAGGGAAGGCAATAAG	MLVA16	[6]
Bruce04-R	CGATCTGGAGATTATCGGGAAG	MLVA16	[6]
Bruce07-F	GCTGACGGGGAAGAACATCTAT	MLVA16	[6]
Bruce07-R	ACCCTTTTTCAGTCAAGGCAAA	MLVA16	[6]
Bruce09-F	GCGGATTCGTTCTTCAGTTATC	MLVA16	[6]
Bruce09-R	GGAGTATGTTTTGGTTGTACATAG	MLVA16	[6]
Bruce16-F	ACGGGAGTTTTTGTTGCTCAAT	MLVA16	[6]
Bruce16-R	GGCCATGTTTCCGTTGATTTAT	MLVA16	[6]
Bruce30-F	TGACCGCAAAACCATATCCTTC	MLVA16	[6]
Bruce30-R	TATGTGCAGAGCTTCATGTTCG	MLVA16	[6]
Bruce18-F	TATGTTAGGGCAATAGGGCAGT	MLVA16	[6]
Bruce18-R	GATGGTTGAGAGCATTGTGAAG	MLVA16	[6]
Bruce19-F	GACGACCCGGACCATGCTT	MLVA16	[6]
Bruce19-R	ACTTCAACCGTAACGTCGTGGAT	MLVA16	[6]
Bruce21-F	CTCATGCGCAACCAAAACA	MLVA16	[6]
Bruce21-R	ATCTCGTGGTCGATAATCTCATT	MLVA16	[6]
